# PUMA Cooperates with p21 to Regulate Mammary Epithelial Morphogenesis and Epithelial-To-Mesenchymal Transition

**DOI:** 10.1371/journal.pone.0066464

**Published:** 2013-06-21

**Authors:** Yanhong Zhang, Wensheng Yan, Yong Sam Jung, Xinbin Chen

**Affiliations:** Comparative Oncology Laboratory, Schools of Medicine and Veterinary Medicine, University of California Davis, Davis, California, United State of America; Vanderbilt University Medical Center, United States of America

## Abstract

Lumen formation is essential for mammary morphogenesis and requires proliferative suppression and apoptotic clearance of the inner cells within developing acini. Previously, we showed that knockdown of p53 or p73 leads to aberrant mammary acinus formation accompanied with decreased expression of p53 family targets PUMA and p21, suggesting that PUMA, an inducer of apoptosis, and p21, an inducer of cell cycle arrest, directly regulate mammary morphogenesis. To address this, we generated multiple MCF10A cell lines in which PUMA, p21, or both were stably knocked down. We found that morphogenesis of MCF10A cells was altered modestly by knockdown of either PUMA or p21 alone but markedly by knockdown of both PUMA and p21. Moreover, we found that knockdown of PUMA and p21 leads to loss of E-cadherin expression along with increased expression of epithelial-to-mesenchymal transition (EMT) markers. Interestingly, we found that knockdown of ΔNp73, which antagonizes the ability of wide-type p53 and TA isoform of p73 to regulate PUMA and p21, mitigates the abnormal morphogenesis and EMT induced by knockdown of PUMA or p21. Together, our data suggest that PUMA cooperates with p21 to regulate normal acinus formation and EMT.

## Introduction

Lumen formation is essential for mammary morphogenesis and requires proliferative suppression and apoptotic clearance of the inner cells within developing acini [1], [2]. Cell proliferation that is not finely balanced by apoptosis may result in accumulation of epithelial cells or premalignant hyperplasia and finally lead to mammary neoplasia [3]. Notably, hallmarks of breast cancer include loss of cell polarity, absence of a hollow lumen, and loss of control of cell proliferation and organization [4]. However, it is still largely unclear what signal pathways directly control the balance between cell proliferation and apoptosis during mammary morphogenesis and tumorigenesis. One of the mechanisms underlying lumen formation might attribute to dynamic expression of the pro-apoptotic factor Bim [5]. Bim is a BH3-only member of the pro-apoptotic BCL-2 family. During in vitro mammary morphogenesis, inhibition of Bim expression significantly decreases apoptotic cell death of the central cells and triggers a filled lumen [5]. Previously, we found that in three-dimensional (3-D) culture of MCF10A mammary epithelial cells, downregulation of wild-type p53 or p73 leads to partial clearance of the inner cells in the lumen due to decreased apoptosis [6], [7]. Since Bim is not a target gene of p53 or p73, it is obvious that in addition to Bim, a p53 family target plays a role in the apoptotic clearance of the inner cells within developing acini.

The p53 upregulated modulator of apoptosis (PUMA), a p53 target, is necessary for stress-induced apoptosis [8], [9]. Like Bim, PUMA is a BH3-only protein of the Bcl-2 family [10], [11]. In addition to its role in tumor suppression, PUMA is involved in development and differentiation of specific tissues and organs. For example, PUMA-induced apoptosis is associated with skeletal myoblast differentiation [12]. Likewise, genetic analysis in Zebrafish revealed that PUMA is essential for development of neural crest-derived lineages during metamorphosis [13]. Recently, we showed that knockdown of p53 or p73 leads to altered acinus formation accompanied with decreased expression of PUMA and p21 [6], [7]. Thus, we hypothesized that loss of PUMA and p21 might disrupt mammary acinus formation via promoting cell proliferation and inhibiting the apoptotic clearance of the inner cells within developing acini. Indeed, we found that p21 and PUMA are necessary for maintaining normal lumen formation and for suppression of epithelial-to-mesenchymal transition (EMT). Additionally, we found that knockdown of ΔNp73 is capable of restoring cell polarity and alleviating EMT induced by knockdown of PUMA or p21.

## Materials and Methods

### Cell Culture

The immortalized MCF10A cell line was obtained from American Type Culture Collection (ATCC, Manassas, VA) and cultured as previously described [6]. The overlay 3-D culture was carried out as described previously with some modifications [6], [14]. Briefly, 4-well chamber slides (Millipore Corporation, Dancers, MA) were pre-coated evenly with 80 µL overnight-thawed Matrigel and MCF10A cells were plated onto Matrigel-coated chamber slides at 5,000 cells/well in complete growth medium with 2% Matrigel and allowed to grow for 1–20 days. Overlay medium containing 2% Matrigel was renewed every 4 days.

### Reagents

Growth factor-reduced Matrigel was purchased from BD Transduction Laboratories (Franklin Lakes, NJ). DMEM/F12 medium, donor horse serum, To-Pro-3 nucleus dye, and anti-mouse antibody conjugated to fluorophore 488 were purchased from Invitrogen (Carlsbad, CA). Hydrocortisone, insulin and cholera toxin were purchased from Sigma (St. Louis, MI). Recombinant human epidermal growth factor (EGF) was purchased from Peprotech (Rocky Hill, NJ). Normal goat serum was purchased from Jackson ImmunoResearch (West Grove, PA).

### Plasmid Constructions and Cell Line Generations

To produce p21 or PUMA shRNA under the control of the U6 promoter, two 62-base oligos were annealed and then cloned into pBabe-U6 shRNA expression vector [6], and the resulting plasmids were designed as pBabe-U6-shp21, or pBabe-U6-shPUMA.

To generate MCF10A cell lines with stable knockdown of ΔNp73 in combination with p21 or PUMA, the following plasmids, pBabe-U6-shΔNp73, pBabe-U6-shp21 or pBabe-U6-shPUMA, were co-transfected into MCF10A cells. The resulting knockdown cell lines were selected with puromycin and confirmed by RT-PCR and/or Western blot analysis. The shRNA oligos used are listed with the siRNA targeting region shown in uppercase (Table. 1).

**Table 1:** The oligos used for generation of shRNA expression vectors.

p21 shRNA1	Sense: 5′-tcgaggtccGCCTCCTCATCCCGTGTTCttcaagagaGAACACGGGATGAGGAGGCtttttg-3′ Antisense: 5′-gatccaaaaaGCCTCCTCATCCCGTGTTCtctcttgaaGAACACGGGATGAGGAGGCggacc-3′
p21 shRNA2	Sense: 5′-tcgaggtccGACCATGTGGACCTGTCACttcaagagaGTGACAGGTCCACATGGTCtttttg-3′ Antisense: 5′-gatccaaaaaGACCATGTGGACCTGTCACtctcttgaaGTGACAGGTCCACATGGTCggacc-3′
PUMA shRNA1	Sense: 5′-tcgaggtccGGGTCCTGTACAATCTCATttcaagagaATGAGATTGTACAGGACCCtttttg-3′ Antisense: 5′-gatccaaaaaGGGTCCTGTACAATCTCATtctcttgaaATGAGATTGTACAGGACCCggacc-3′
PUMA shRNA2	Sense: 5′-tcgaggtccGCCTGTAAGATACTGTATAttcaagagaTATACAGTATCTTACAGGCtttttg-3′ Antisense: 5′-gatccaaaaaGCCTGTAAGATACTGTATATtctcttgaaTATACAGTATCTTACAGGCggacc-3′
ΔNp73 shRNA1	Sense: 5′-tcgaggtccGACAGAACTAAGGGAGATGttcaagagaCATCTCCCTTAGTTCTGTCtttttg-3′ Antisense: 5′-gatccaaaaaGACAGAACTAAGGGAGATGtctcttgaaCATCTCCCTTAGTTCTGTCggacc-3′
ΔNp73 shRNA2	Sense: 5′-tcgaggtccGGATTCAGCCAGTTGACAGttcaagagaCTGTCAACTGGCTGAATCCtttttg-3′ Antisense: 5′-gatccaaaaaGGATTCAGCCAGTTGACAGtctcttgaaCTGTCAACTGGCTGAATCCggacc-3′

### RT-PCR Analysis

Total RNA was extracted from cells using TRIzol (Invitrogen Life Technoloogies, Inc.) according to manufacturer’s instructions. cDNA was synthesized using M-MLV Reverse Transcriptase Kit (Promega Corporation) according to manufacturer’s protocol. The primers to detect ΔNp73 are sense 5′-gatccatgccctcgtcccac-3′ and antisense 5′-ctgctcatctggtccatgg-3′. The *Actin* gene was chosen as a loading control and detected with primers 5′-ctgaagtaccccatcgagcacggca-3′ (sense) and 5′-ggatagcacagcctggatagcaacg-3′ (antisense).

### Western Blot Analysis

Western blotting was performed as described [15]. Antibodies used were purchased from Millipore (Temecula, CA; anti-laminin γ2), Santa Cruz Biotechnology (anti-β-catenin (E-5), anti-Snail-1, anti-Twist, and anti-p21 (H-164)), Cell Signaling (anti-Slug), BD Transduction Laboratories (anti-E-cadherin), Calbiochem (p73 (ab-3)), ProSci incorporated (anti-PUMA), Sigma (anti-actin), and BioRad (Life Science Research, Hercules, CA; secondary antibodies against rabbit or mouse IgG conjugated with HRP).

### Confocal Microscopy

3-D structures in Matrigel were fixed in 4% paraformaldehyde at room temperature for 20 min and permeabilized with 0.5% Triton-X100 in PBS for 30 min at 4°C. After quenching with 100 mM glycine in PBS, the structures were pre-blocked in a primary blocking buffer A (130 mM NaCl, 7 mM Na_2_HPO_4_, 3.5 mM NaH_2_PO_4_, 0.1% BSA, 0.2% Triton X-100 and 0.05% Tween-20) containing 10% normal goat serum for 2 h and further blocked in a secondary blocking buffer B (buffer A plus 10% normal goat serum and 20 mg/mL of goat anti-mouse F[ab′]_2_ fragments) for 1 h. The structures were incubated overnight with primary antibodies at 4°C, washed thoroughly with buffer A with gentle shaking, and stained with FITC-conjugated secondary antibody (diluted 1∶200 in buffer A) for 1 h. The structures were nuclear stained with 5 µg/mL of To-Pro-3 in PBS for 15 min at room temperature. The To-Pro-3 stain was removed by washing the chamber slide with PBS for 5 min and then the slide was mounted under a glass cover slip with 0.1% para-phenylenediamine D (PPD) and 90% glycerol in PBS. Microscopic analysis was performed using a confocal microscopy system (Axiovert 100 m, Zeiss) and images were acquired using the software for Carl Zeiss Laser Scanning Microscope (LSM-510). The images of acinus structures were captured by the Z-stacking function for serial confocal sectioning at 2 µm intervals. Experiments were conducted in triplicate.

### Colony Formation Assay

MCF10A cells were cultured in a 6-well plate for ∼12 days and then fixed with methanol/glacial acetic acid (7∶1) followed by staining with 0.1% crystal violet. Experiments were performed in triplicate.

### *In vitro* Cell Migration Assay

For wound healing assay, cells were grown in a 6-well plate for 24 h. The monolayers were wounded by scraping with a P200 micropipette tip and washed two times with PBS. At specified time points after the scraping, cell monolayers were photographed using a Canon EOS 40D digital camera (Canon, Lake Success, NY). Migration rate of cells was measured by averaging the time required to close the borders of cells. Six regions were analyzed in each well, and the result was expressed as the mean ± SD. Experiments were conducted in triplicate.

### Statistical Analysis

Data were presented as Mean ± SD. Statistical significance was determined by Student’s t test. Values of P<0.05 were considered significant.

## Results

### PUMA is Necessary for Morphogenesis of MCF10A Cells

**Figure 1:**
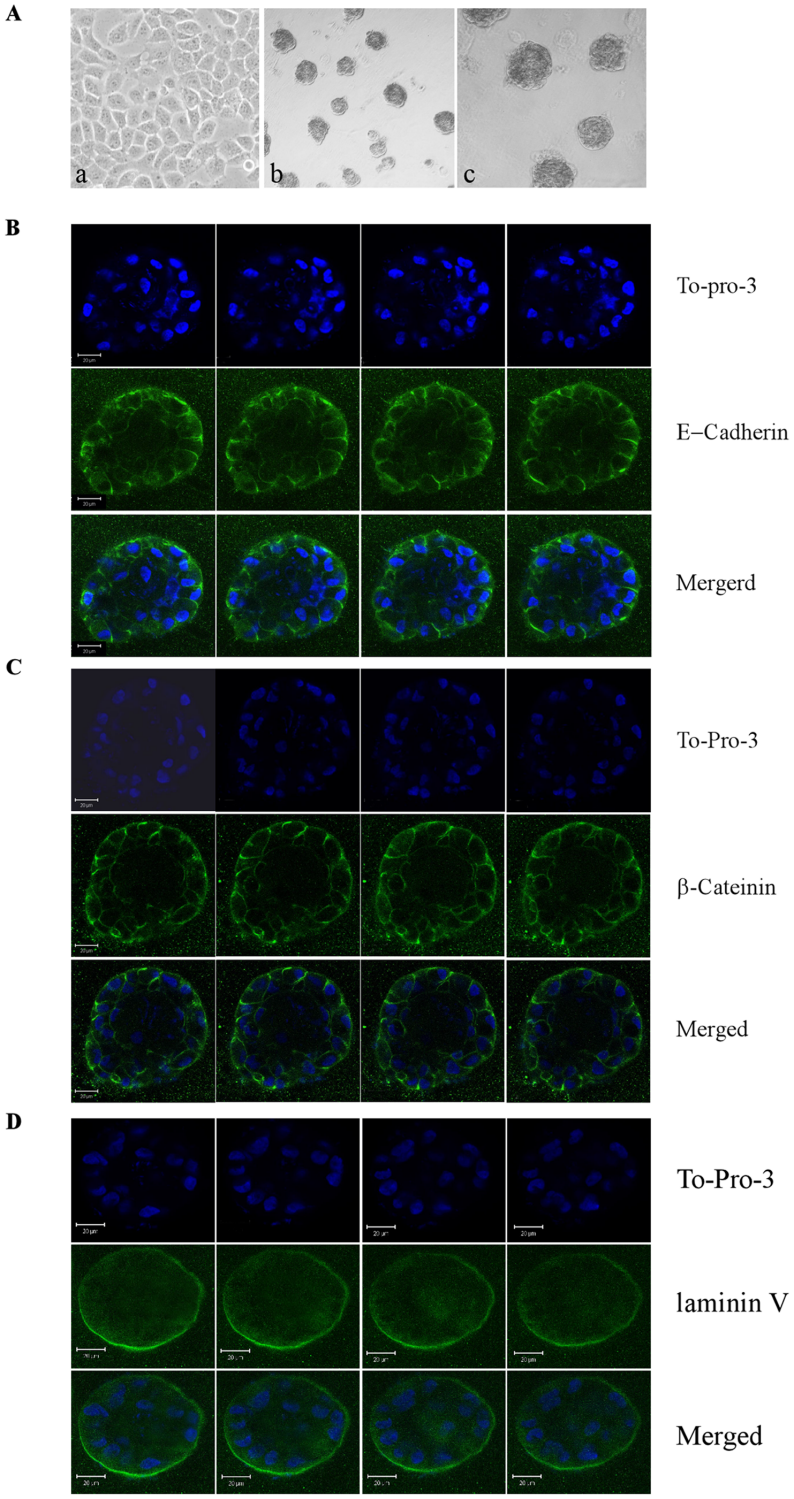
Mammary epithelial cells cultured on ECM form functional acini. **A,** Representative phase-contrast microscopic images of MCF10A cells in 2-D culture (a, 200×) and 3-D culture (b, 40×; c, 100×). **B,** Serial confocal images of cross-sections through the middle of acini stained with To-Pro-3 and antibody against E-cadherin in MCF10A cells. **C,** Serial confocal images of cross-sections through the middle of acini stained with To-Pro-3 and antibody against β-catenin in MCF10A cells. **D**, Serial confocal images of cross-sections through the middle of acini stained with To-Pro-3 and antibody against laminin V in MCF10A cells. Scale bar, 20 µm.

**Figure 2:**
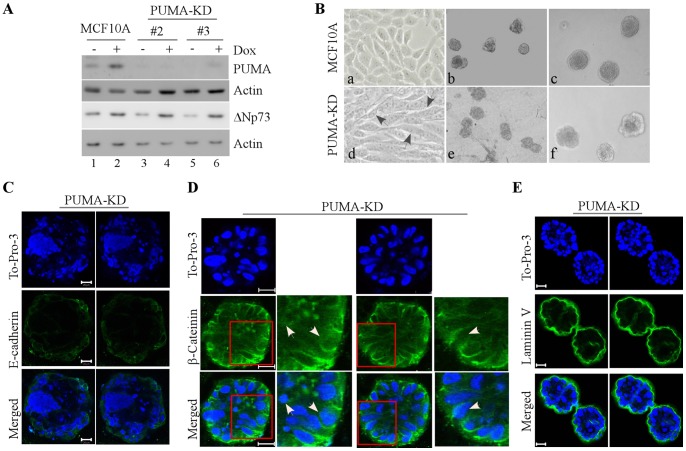
PUMA is necessary for morphogenesis of MCF10A cells. **A**, Generation of MCF10A cells in which PUMA (clones #2 and 3) was stably knocked down. Western blots were performed with extracts from MCF10A cells untreated or treated with 0.2 µM doxorubicin for 24 h and then probed with antibodies against PUMA, ΔNp73 and actin, respectively. **B,** Representative images of MCF10A cells or MCF10A cells with PUMA-KD in 2-D culture (a and d, 200×) and 3-D culture (b and e, 40×; c and f, 100×). Black arrow indicates elongated spindle–liked MCF10A cells. **C,** Representative confocal images of cross-sections through the middle of acini stained with To-Pro-3 and antibody against E-cadherin in MCF10A cells with PUMA-KD. **D,** Representative confocal images of cross-sections through the middle of acini stained with To-Pro-3 and antibody against β-catenin in MCF10A cells with PUMA-KD. White arrows indicate the accumulation and translocation of β-catenin in acinus structure. **E**, Representative confocal images of cross-sections through the middle of acini stained with To-Pro-3 and antibody against laminin V in MCF10A cells with PUMA-KD. Scale bar, 20 µm.

MCF10A cells in 3-D culture undergo various biological events, such as apoptosis, proliferative suppression, polarization and cell adhesion, to form an acinus structure with a hollow lumen similar to the normal acinus *in vivo* [1], [2]. Consistently, we showed that MCF10A cells exhibited normal cobble-stone-like epithelial cell morphology in 2-D culture (Figure 1A, a) and formed acinus-like structures in 3-D culture (Figure 1A, b and c) along with hollow lumen (Figure 1B-D). In addition, cells showed an apical-basal distribution of polarity marker laminin V and cell-cell junction markers, such as E-cadherin and β-catenin (Figure 1B–D). Previously, we showed that knockdown of p53 or p73 in 3-D cultured MCF10A cells disrupts acinus structure coupled with decreased levels of p53 family targets PUMA and p21 [6], [7]. PUMA is a pro-apoptotic BH3 protein [10] and mediates apoptosis [12], whereas p21 mediates p53 family-dependent cell cycle arrest [16]. However, it is unclear whether PUMA or p21 plays a role in morphogenesis of mammary epithelial cells. To test this, we generated multiple MCF10A cell lines in which PUMA was stably knocked down by shRNA (Figure 2A, clones #2–3). We showed that in parental MCF10A cells, PUMA was induced upon treatment of doxorubicin (Figure 2A, compare lane 1 vs. 2), whereas in cells with PUMA knockdown (PUMA-KD), the levels of PUMA were decreased by shRNA at both the basal and stress conditions (Figure 2A, lanes 3–6). We found that PUMA knockdown had little if any effect on the expression of ΔNp73 in MCF10A cells (Figure 2A, compare lanes 3–6 to lanes 1–2), although the level of ΔNp73 was increased upon doxorubicin treatment. We also found that compared to MCF10A cells, MCF10A cells with PUMA-KD exhibited elongated morphology in 2-D culture (Figure 2B, a and d) and irregular and near-normal spheroids in 3-D culture (Figure 2B, b–c and e–f). In addition, we found that in 3-D culture, PUMA-KD MCF10A cells formed acini with partially filled lumen (Figure 2C–E), which are different from the acini with hollow lumen formed by parental MCF10A cells. Furthermore, we found that PUMA-KD MCF10A cells exhibited weak E-cadherin staining at the periphery of acini (Figure 2C), strong β-catenin staining at the cell-cell junction (Figure 2D), and near-normal laminin V staining at the basal membrane (Figure 2E).

### p21 is Necessary for Morphogenesis of MCF10A Cells

**Figure 3:**
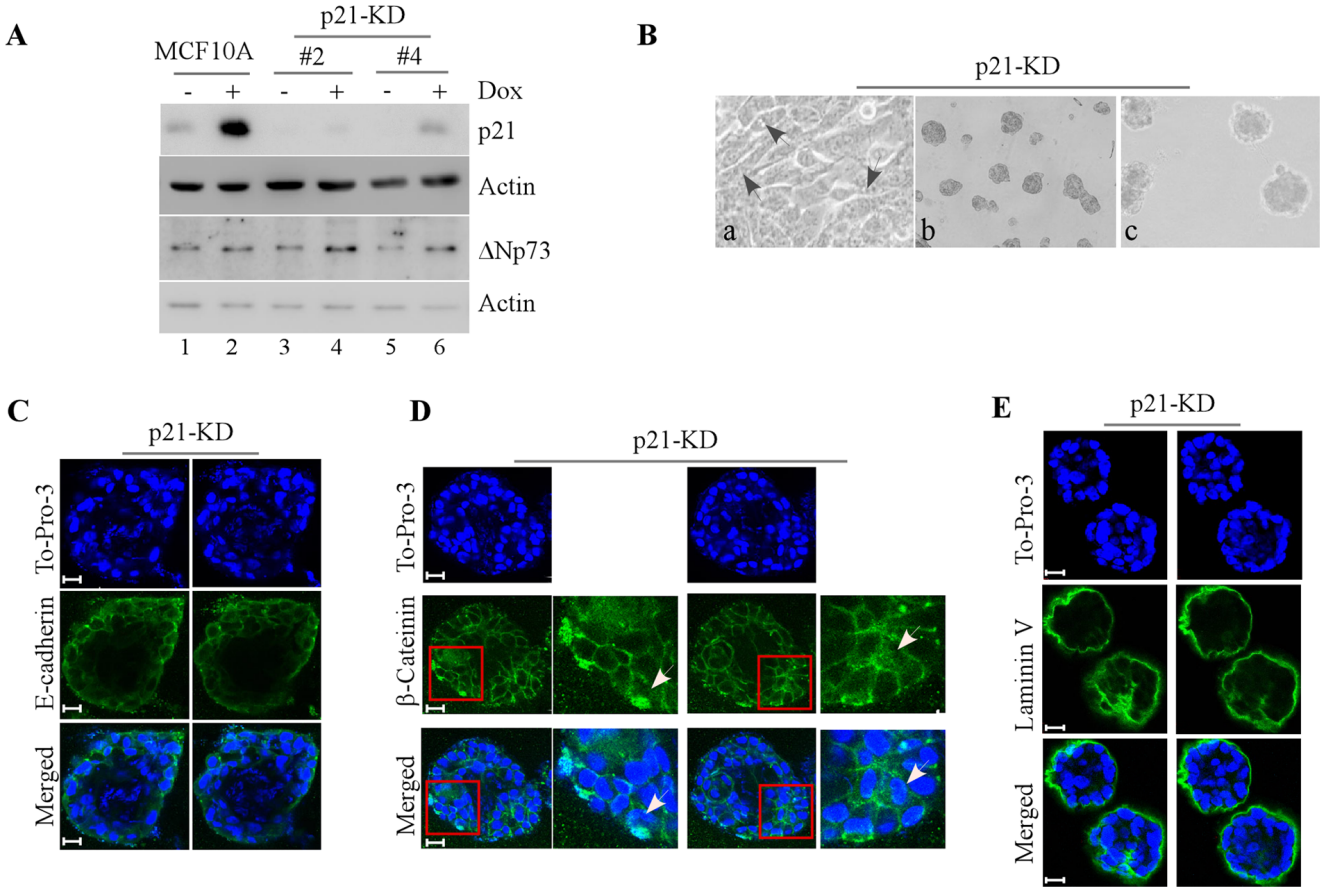
p21 is necessary for morphogenesis of MCF10A cells. **A,** Generation of MCF10A cells in which p21 was stably knocked down (clones #2 and #4). Western blot were performed with extracts from MCF10A cells untreated or treated with 0.2 µM doxorubicin for 24 h and then probed with antibodies against p21, ΔNp73 and actin, respectively. **B,** Representative phase-contrast microscopic images of MCF10A cells with p21-KD in 2-D culture (a, 200×,) and 3-D culture (b, 40×, and c, 100×). Black arrow indicates elongated spindle–liked MCF10A cells. **C,** Representative confocal images of cross-sections through the middle of an acinus stained with To-Pro-3 and antibody against E-cadherin. **D,** Representative confocal images of cross-sections through the middle of acini stained with To-Pro-3 and antibody against β-catenin. White arrows indicate the accumulation and translocation of β-catenin in an acinus structure. **E,** Representative confocal images of cross-sections through the middle of an acinus stained with To-Pro-3 and antibody against laminin V. Scale bar, 20 µm.

Next, to examine the role of p21 in mammary morphogenesis, we generated multiple MCF10A cell lines in which p21 was stably knocked down by shRNA (Figure 3A, clones #2 and #4). We showed that in parental MCF10A cells, p21 was induced upon treatment of doxorubicin (Figure 3A, compare lane 1 vs. 2). However, upon p21 knockdown (p21-KD), the levels of p21 protein were decreased by shRNA at both the basal and stress conditions (Figure 3A, lanes 3–6). In addition, we found that the level of ΔNp73 in MCF10A cells, which was induced by treratment of doxorubicin, was not obviously affected by p21 knockdown (Figure 3A, compare lanes 3–6 to lanes 1–2). Furthermore, we found that similar to PUMA-KD MCF10A cells, p21-KD cells exhibited an elongated morphology in 2-D culture (Figure 3B, a), irregular and near-normal spheroids in 3-D culture (Figure 3B, b–c), partially filled lumen (Figure 3C–E), weak E-cadherin staining at the periphery of acini (Figure 3C), strong β-catenin staining at the cell-cell junction (Figure 3D), and a near-normal laminin V staining at the basal membrane (Figure 3E).

### PUMA Cooperates with p21 to Regulate Morphogenesis of MCF10A Cells

**Figure 4:**
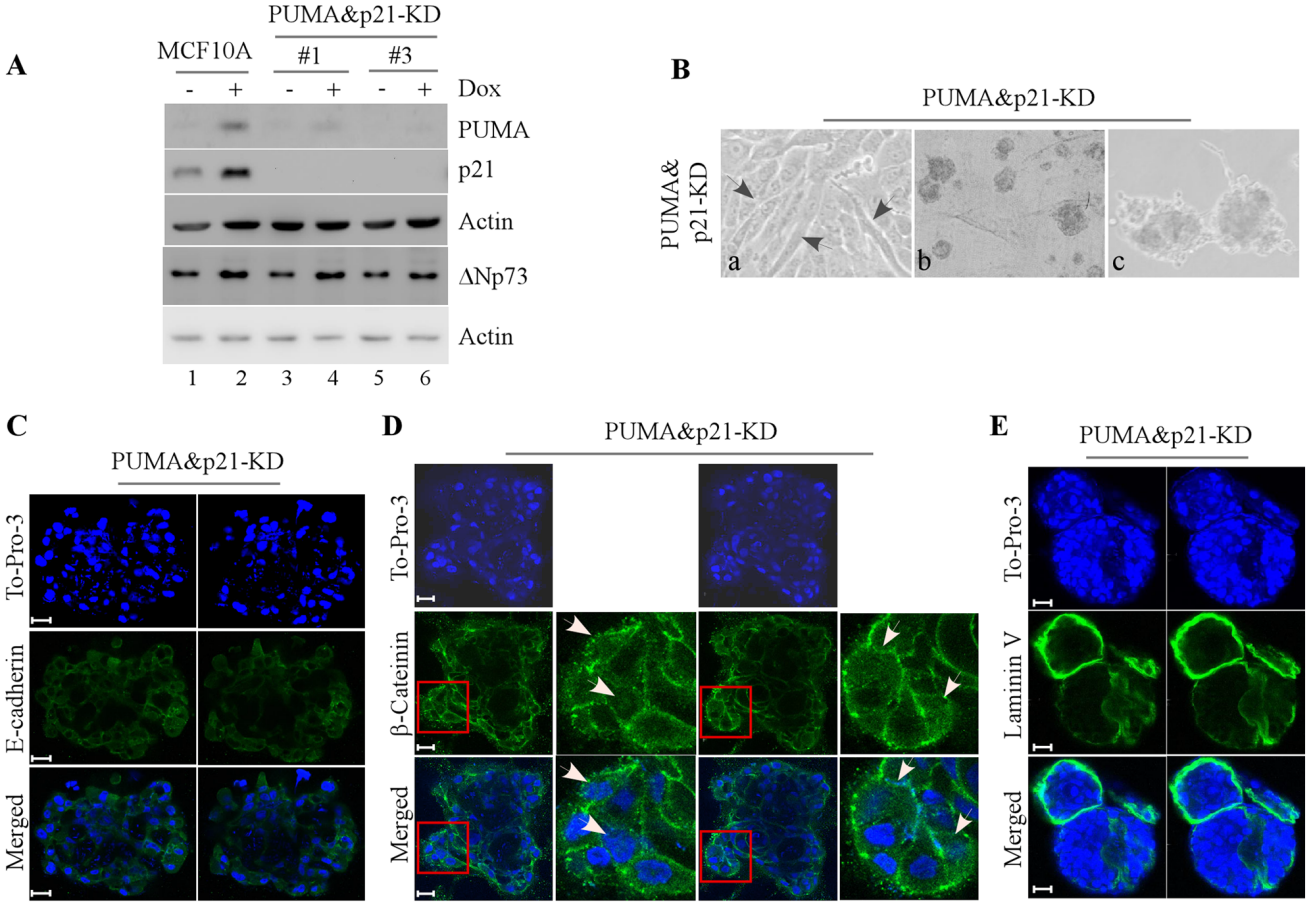
PUMA cooperates with p21 to regulate morphogeneis of MCF10A cells. **A**, Generation of MCF10A cells in which both PUMA and p21 were stably knocked down (clone #1 and #3). Western blots were prepared with extracts from MCF10A cells untreated or treated with 0.2 µM doxorubicin for 24 h and then probed with antibodies against PUMA, p21, ΔNp73, and actin, respectively. **B,** Representative images of MCF10A cells with PUMA&p21-KD in 2-D culture (a, 200×) and 3-D culture (b, 40×; c, 100×). Black arrow indicates elongated spindle-liked MCF10A cells. **C,** Representative confocal images of cross-sections through the middle of an acinus stained with To-Pro-3 and antibody against E-cadherin. **D,** Representative confocal images of cross-sections through the middle of acini stained with To-Pro-3 and antibody against β-catenin. White arrows indicate the accumulation and translocation of β-catenin in an acinus structure. **E,** Representative confocal images of cross-sections through the middle of an acinus stained with To-Pro-3 and antibody against laminin V. Scale bar, 20 µm.

The above observations suggest that lack of PUMA or p21 leads to partial lumen clearance but is not sufficient to completely disrupt morphogenesis of MCF10A cells. Thus, we explored whether PUMA cooperates with p21 to regulate morphogenesis of MCF10A cells. To test this, we generated MCF10A cell lines in which both PUMA and p21 were stably knocked down (Figure 4A, clones #1 and #3). We showed that in parental MCF10A cells, PUMA and p21 were induced upon treatment of doxorubicin (Figure 4A, compare lanes 1–2). However, in MCF10A cells with knockdown of PUMA and p21 (PUMA&p21-KD), the levels of both p21 and PUMA were decreased by shRNAs at the basal and stress conditions (Figure 4A, lanes 3–6). In addition, we found that the level of ΔNp73, which was induced by treatment of doxorubicin, was not obviously altered upon knockdown of both PUMA and p21 (Figure 4A, compare lanes 3–6 to lanes 1–2). We also showed that upon PUMA&p21-KD, MCF10A cells exhibited remarkable spindle-like morphology in 2-D culture (Figure 4B, a) and irregular and multi-acinus structures in 3-D culture (Figure 4B, b–c). Moreover, we found that the lumen of acini was not cleared (Figure 4C–E) and that the staining pattern of E-cadherin became scattered and non-polarized (Figure 4C). Furthermore, we found that β-catenin was mainly expressed in the nuclear and cytosol (Figure 4D) and that laminin V was deposited at the apical-basal junction as well as at the periphery of the filled lumen (Figure 4E). These observations suggest that lack of both PUMA and p21 completely disrupts normal cell polarity and tight junction of MCF10A cells.

### Knockdown of PUMA and p21 Promotes EMT

**Figure 5:**
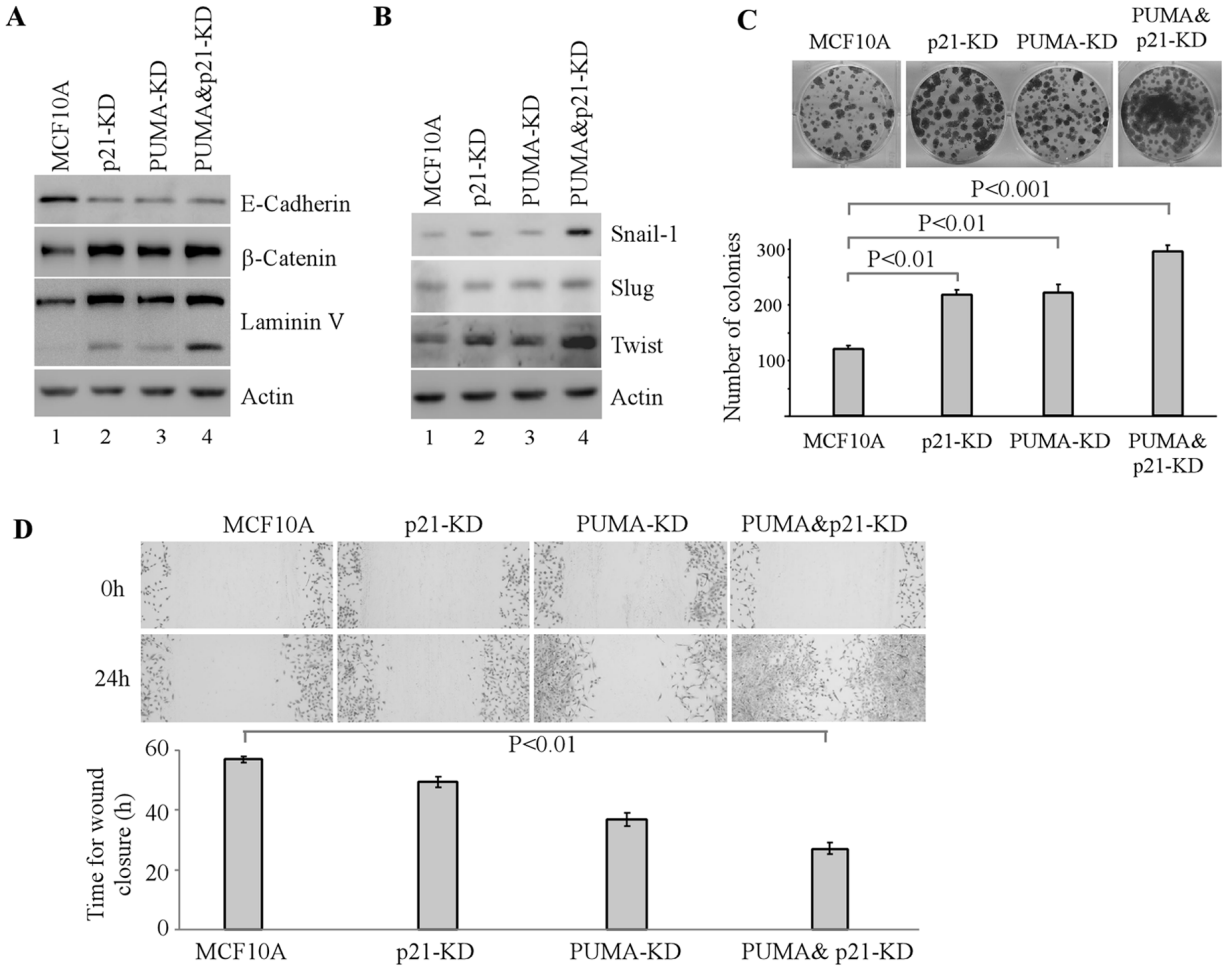
Knockdown of PUMA and p21 enhances EMT. **A-B**, Western blots were prepared with extracts from MCF10A cells (lane 1), and MCF10A cells with p21-KD (lane 2), PUMA-KD (lane 3), or PUMA&p21-KD (lane 4). MCF10A cells were grown in Matrigel for 20 days. The blots were probed with antibodies against E-cadherin (A), β-catenin (A), laminin V (A), Snail-1 (B), Slug (B), Twist (B), and actin (A–B), respectively. **C,** Top panel: Colony formation assay was performed with MCF10A cells, or MCF10A cells with p21-KD, PUMA-KD or PUMA&p21-KD. Cells were cultured for a period of 12 days, then fixed and stained with crystal violet. Bottom panel: The number of colonies was counted and presented as Mean ± SD from three separate experiments. **D,** Top panel: Wound healing assay was performed with MCF10A cells and MCF10A cells with p21-KD, PUMA-KD or PUMA&p21 -KD. Cell migration was determined by visual assessment of cells migrating into the wound for a period of 24 h using a phase-contrast microscopy. Bottom panel: The time required for wound closure was measured and presented as Mean ± SD from three separate experiments.

Loss of cell polarity induces EMT [17]. Thus, we examined whether knockdown of PUMA and p21 leads to EMT. We found that upon knockdown of p21 and/or PUMA, the levels of β-catenin and laminin V were increased whereas the level of E-cadherin was decreased (Figure 5A), which is consistent with altered staining patterns of these EMT markers in acinus-like structures (Figures 2–4, C-E). In addition, we found that Snail-1, Twist and to lesser extent Slug were highly induced by PUMA&p21-KD, but only mildly induced by p21-KD or PUMA-KD individually (Figure 5B). Consistently, colony formation and wound healing assays showed that cell proliferation and migration were highly increased by PUMA&p21-KD compared to p21-KD or PUMA-KD alone (Figure 5C–D). Together, these findings suggest that PUMA&p21-KD disrupts cell polarity and acinus formation and leads to EMT.

### Knockdown ofΔNp73 Counters the Effect of PUMA-KD or p21-KD on MCF10A Cell Polarity

**Figure 6:**
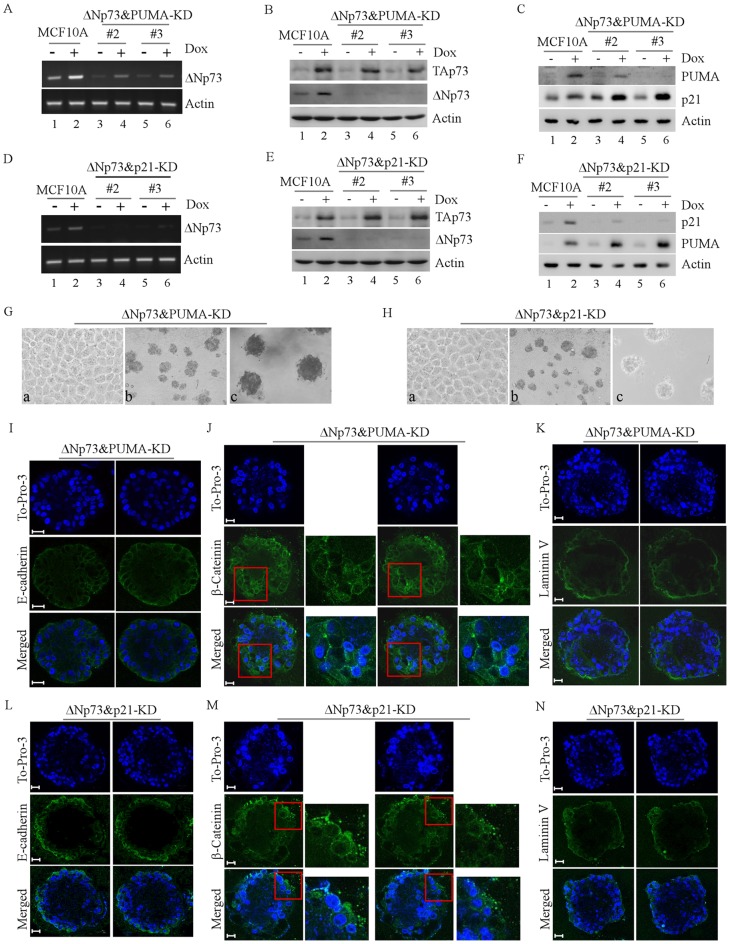
Knockdown ofΔNp73 counters the effect of PUMA-KD or p21-KD on MCF10A cell morphogenesis. **A-F,** Generation of MCF10A cells in which both ΔNp73 and PUMA were stably knocked down (A-C, clones #2 and #3) or ΔNp73 and p21 were stably knocked down (D-F**,** clones #2 and #3). The levels of ΔNp73 mRNA were measured by RT-PCR (A and D). The protein levels of TAp73α (B and E), ΔNp73α (B and E), PUMA (C and F), and p21 (C and F) were measured by Western blotting with antibodies against TAp73, ΔNp73, p21, and PUMA, respectively. MCF10A cells were untreated or treated with 0.2 µM doxorubicin for 24 h and total RNAs and cell extracts were collected for RT-PCR and Western blotting, respectively. **G-H**, Representative images of MCF10A cells with ΔNp73& PUMA -KD (G) or with ΔNp73&p21-KD (H) in 2-D culture (a, 200×) and 3-D culture (b, 40×; c, 100×). **I** and **L,** Representative confocal images of cross-sections through the middle of acini stained with To-Pro-3 and antibody against E-cadherin in MCF10A cells with ΔNp73&PUMA -KD (I) or with ΔNp73&p21 -KD (L). **J** and **M,** Representative confocal images of cross-sections through the middle of acini stained with To-Pro-3 and antibody against β-catenin in MCF10A cells with ΔNp73&PUMA-KD (J) or with ΔNp73&p21-KD (M). **K** and **N,** Representative confocal images of cross-sections through the middle of acini stained with To-Pro-3 and antibody against laminin V in MCF10A cells with ΔNp73&PUMA-KD (K) or with ΔNp73&p21-KD (N). Scale bar, 20 µm.

Here, we found that PUMA-KD or p21-KD led to irregular acinus-like structures with filled lumen (Figures 2–3). Previously, we showed that knockdown of ΔNp73 (ΔNp73-KD) leads to increased expression of p21 and PUMA and subsequently decreased cell proliferation in MCF10A cells [7]. It is worth to mention that ΔNp73 is not only dominant-negative over TAp73 but also has its own distinct activity [18], [19]. Thus, we examined whether ΔNp73-KD counters the effect of PUMA-KD or p21-KD on cell polarity in MCF10A cells. To test this, we generated MCF10A cells in which ΔNp73 and PUMA (Figure 6A–C: ΔNp73&PUMA-KD) or ΔNp73 and p21 (Figure 6D–F: ΔNp73&p21-KD) were simultaneously knocked down. We showed that in parental MCF10A cells, doxorubicin treatment induced both ΔNp73 and TAp73 (Figure 6A–B, and D-E, compare lane 1 vs. 2), consistent with the previous reports [18], [20], [21]. In addition, we showed that in MCF10A cells, only ΔNp73, but not TAp73, was knocked down by shRNA against ΔNp73 (Figures 6, A–B and D–E, lanes 3–6). Furthermore, we found that in ΔNp73&PUMA-KD cells, the level of PUMA was decreased by PUMA shRNA whereas the level of p21 was increased upon knockdown of ΔNp73 regardless of doxorubicin treatment (Figure 6C, lanes 3–6). Likewise, we found that in ΔNp73&p21-KD cells, the level of p21 was decreased by p21 shRNA but the level of PUMA was increased upon knockdown of ΔNp73 (Figure 6F, lanes 3–6).

Next, we tested whether ΔNp73 counters the effect of PUMA-KD or p21-KD on acinus formation. We found that MCF10A cells with Np73&PUMA-KD exhibited normal cobble-stone-like epithelial cell morphology in 2-D culture (Figure 6G, a) and formed regular spheroids in 3-D culture (Figure 6G, b–c) along with near-hollow lumen (Figure 6I–K). In addition, we found that MCF10A cells with ΔNp73&PUMA-KD exhibited near-normal staining patterns for E-cadherin (mostly at cell-cell junctions) (Figure 6I) and laminin V staining (mostly apical-basal deposition) (Figure 6K), but a small increase in β-catenin (mostly polarized lateral distribution) (Figure 6J). Moreover, we found that MCF10A cells with ΔNp73&p21-KD exhibited similar phenotypes as ΔNp73&PUMA-KD cells (Figure 6H and I–N). Nevertheless, these phenotypes exhibited by cells with ΔNp73&PUMA-KD and ΔNp73&p21-KD are markedly different from that exhibited by cells with PUMA-KD (Figure 2C–E) and p21-KD alone (Figure 3C–E), suggesting that knockdown of ΔNp73 is able to counter the abnormal morphogenesis of MCF10A cells induced by PUMA-KD or p21-KD.

**Figure 7:**
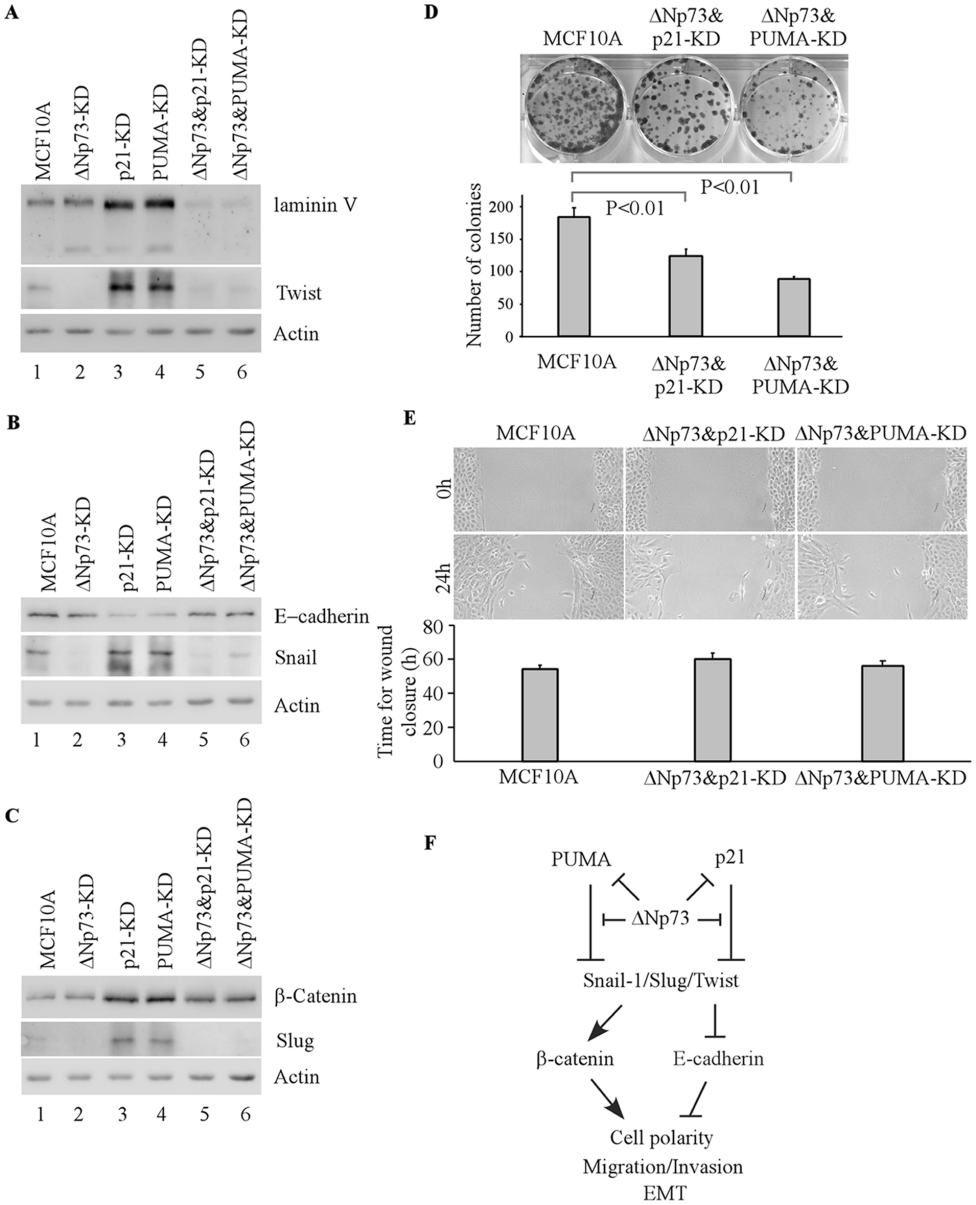
Knockdown ofΔNp73 mitigates EMT induced by PUMA-KD or p21-KD. **A–C**,MCF10A cells were grown in Matrigel for 20 days. Western blots were prepared using extracts from MCF10A cells (lane 1), MCF10A cells with ΔNp73-KD (lane 2), with p21-KD (lane 3), with PUMA-KD (lane 4), with ΔNp73&p21-KD (lane 5) or with ΔNp73&PUMA-KD (lane 6). The blots were probed with antibodies against laminin V (A), Twist (A), E-cadherin (B), Snail-1 (B), β-catenin (C), Slug (C), and actin (A-C), respectively. **D,** Top panel: Colony formation assay was performed with MCF10A cells and MCF10A cells with ΔNp73&p21-KD or with ΔNp73&PUMA-KD. Bottom panel: the number of colonies was counted and presented as Mean ± SD from three separate experiments. **E,** Top panel: Wound healing assay was performed with MCF10A cells and MCF10A cells with ΔNp73&p21-KD or with ΔNp73&PUMA-KD. Cell migration was determined by visual assessment of cells migrating into the wound for a period of 24 h using a phase-contrast microscopy. Bottom panel: The time required for wound closure was measured and presented as Mean ± SD from three separate experiments. **F,** A model of PUMA, p21 and ΔNp73 in cell polarity.

Next, we examined whether ΔNp73-KD counters the effect of PUMA-KD or p21-KD on EMT. We found that the expression levels for E-cadherin were nearly restored to near-normal levels, whereas the levels for β-catenin and laminin V were decreased, in MCF10A cells upon knockdown of ΔNp73&PUMA or ΔNp73&p21 as compared to the cells upon knockdown of p21 or PUMA alone (Figure 7A–C). In addition, knockdown of ΔNp73&PUMA or ΔNp73&p21 led to decreased expression of Snail-1, Slug, and Twist in MCF10A cells (Figure 7A–C). This suggests that ΔNp73-KD counters the effect of PUMA-KD or p21-KD on expression of laminin V, β-catenin, E-cadherin, Snail-1, Slug, and Twist (Figure 5A–B). Next, colony formation assay was performed and showed that cell proliferation was inhibited upon ΔNp73&PUMA-KD or ΔNp73&p21-KD (Figure 7D), suggesting that ΔNp73-KD blocks enhanced cell proliferation by PUMA-KD or p21-KD (Figure 5C). Finally, wound healing assay was performed and showed that upon knockdown of ΔNp73&PUMA or ΔNp73&p21, cell migration was slightly suppressed compared to that in control cells (Figure 7E), suggesting that ΔNp73-KD blocks slightly enhanced cell migration by p21-KD or PUMA-KD (Figure 5D). Taken together, these observations suggest that ΔNp73-KD is capable of mitigating the effect of p21-KD or PUMA-KD on cell polarity and EMT.

## Discussion

Mammary gland is an intact, well-ordered architecture. One event during mammary morphogenesis is acinus formation. This process starts with establishment of epithelial cell polarity, which then modulates cell proliferation and apoptosis required for acinus maturation and lumen formation [1], [4]. Previously, we found that downregulation of wild-type p53 or p73 leads to disruption of acinus formation and decreased expression of their target genes, including p21 and PUMA [6], [7]. It is well-known that PUMA causes cell death via apoptosis and p21 inhibits cell proliferation via cell cycle arrest. However, p21 and PUMA may also play a role in development of specific tissues and organs [12], [13], [22]–[26]. Here, we utilized the 3-D culture model of MCF10A cells to examine the role of PUMA and p21 in the morphogenesis of mammary epithelial cells. Interestingly, we found that MCF10A cells with knockdown of PUMA or p21 alone still form near-normal spheroids but with incomplete lumen clearance (Figures 2–3). Moreover, knockdown of both PUMA and p21 interferes with distribution of polarity-tight junction markers along with formation of multi-acinar spheroids (Figure 4). These observations suggest that during acinus morphogenesis, PUMA is involved in the clearance of inner cells while p21 suppresses abnormal cell proliferation in the lumen. This result recapitulates the phenotype of cell polarity altered by knockdown of wild-type p53 or TAp73 [6], [7], suggesting that p21 and PUMA function downstream of wild-type p53 and p73 to maintain normal epithelial morphogenesis. In addition, our study is consistent with the recent report, which showed that PUMA/p21 double knockout mice have a phenotype similar to p53 knockout mice upon lethal irradiation, with blocked apoptosis but exacerbated gastro-intestinal epithelial damage [27]. Thus, loss of genes that regulate cell proliferation and apoptosis may lead to tumorigenesis in the mammary gland. Our observations support the postulation that both anti-proliferation and apoptotic activities are required for achieving lumen formation in mammary epithelial acini (Figure 7F).

EMT plays an important role in embryogenesis and development. During EMT, epithelial cells lose their epithelial features and acquire a fibroblast-like morphology, accompanied with up-regulation of mesenchymal markers and enhancement of migratory properties, contributing to pathological processes such as fibrosis and cancer [28], [29]. EMT is triggered by diverse signal pathways, including transforming growth factor-β (TGF-β), Wnt, Hedgehog, and Notch [30]. Previous study showed that p21 is responsible for preventing TGF-β from inducing cell proliferation in MCF10A cells [31]. Furthermore, TGF-β confers p21-null cells to mesenchymal transition with increased expression of vimentin and decreased expression of E-cadherin [32]. In addition, loss of p21 enhances, whereas ectopic expression of p21 represses, the features of EMT in transformed human mammary epithelial cell lines [33]. Moreover, p21 prevents Twist transcription factor from repressing E-cadherin expression [33]. Importantly, loss of p21 is correlated with positive vimentin expression in primary human breast cancers [32]. Here, we found that upon knockdown of p21, PUMA and especially both, MCF10A cells undergo EMT and exhibit loss of E-cadherin expression, accumulation of β-catenin in the nucleus, increased expression of laminin V and up-regulated EMT markers (Snail-1, Slug and Twist). In line with this, we observed that combined knockdown of p21 and PUMA leads to formation of acini with filled lumen and acquisition of enhanced migratory activity (Figure 5). These results further confirm the role of p21 in EMT, but most importantly, uncover a novel function for PUMA as a determinant of EMT in the morphogenesis of mammary epithelial cells. It is known that Slug is a suppressor of PUMA [34] and knockdown of Slug promotes apoptosis by up-regulation of PUMA [35], [36]. Here, we found that PUMA-KD increases the expression of Slug. Thus, the mutual regulation between PUMA-KD and Slug upregulation represents a novel feed-forward loop. We postulate that in response to downregulation of PUMA, Slug expression is induced, which in turn further inhibits expression of PUMA. As a result, the signaling cascade for EMT is amplified. In addition, we found that the levels of EMT markers (Snail-1, Slug and Twist) increased by knockdown of both p21 and PUMA are much higher than that by p21-KD and PUMA-KD alone. Moreover, the EMT morphology is profound in the cells with p21&PUMA-KD. In light of these observations, we speculate that PUMA and p21 are two important determinants for EMT in the aberrant morphogenesis of mammary epithelial cells, and that PUMA might cooperate with p21 to prevent EMT in mammary epithelial cells via repressing expression of these transcription factors.

ΔN isoform of p73 possesses a dominant negative activity towards TAp73 and possibly p53 [37], [38]. Overexpression of ΔNp73 downregulates target genes of TAp73 and wild-type p53, such as the death receptors CD95 and TRAIL-R2 [39]. Conversely, deficiency of ΔNp73 leads to increased expression of p21 and PUMA [7], [40], [41]. Significantly, inactivation of ΔNp73 was found to increase apoptosis in mouse brain development [41], [42]. Here, we found that in ΔNp73&PUMA-KD cells, knockdown of ΔNp73 mitigates the effect of PUMA-KD on cell polarity and EMT. This may be partly because p21 expression is increased by ΔNp73-KD. Similarly, in ΔNp73&p21-KD cells, ΔNp73-KD increases PUMA expression to compensatorily alleviate EMT induced by p21-KD. Since ΔNp73 has its own distinct activities [18], [19], the counteracting effect of ΔNp73-KD on EMT may be due to the fact that ΔNp73 is required for increased expression of the EMT inducers (Snail-1, Slug, and Twist) (Figure 7A–C).
