# Correction: PUMA Cooperates with p21 to Regulate Mammary Epithelial Morphogenesis and Epithelial-To-Mesenchymal Transition

**DOI:** 10.1371/journal.pone.0237624

**Published:** 2020-08-07

**Authors:** Yanhong Zhang, Wensheng Yan, Yong Sam Jung, Xinbin Chen

During the figure preparation of this article [1], the ΔNp73 panel of Fig 6B representing ΔNp73&PUMA-KD was inadvertently used for ΔNp73 in Fig 6E representing ΔNp73&p21-KD. The updated version of Fig 6E shows the correct panel for ΔNp73. The underlying blots for Fig 6B and 6E confirming the results have been uploaded as S1–S3 Files. The TAp73 and Actin panels derive from the same gel, whereas the ΔNp73 panels were run on a separate gel. The legend of Fig 6 has been updated to reflect this. The authors would like to clarify that the samples presented in Fig 6B and Fig 6E were collected from independent experiments.

**Fig 6:**
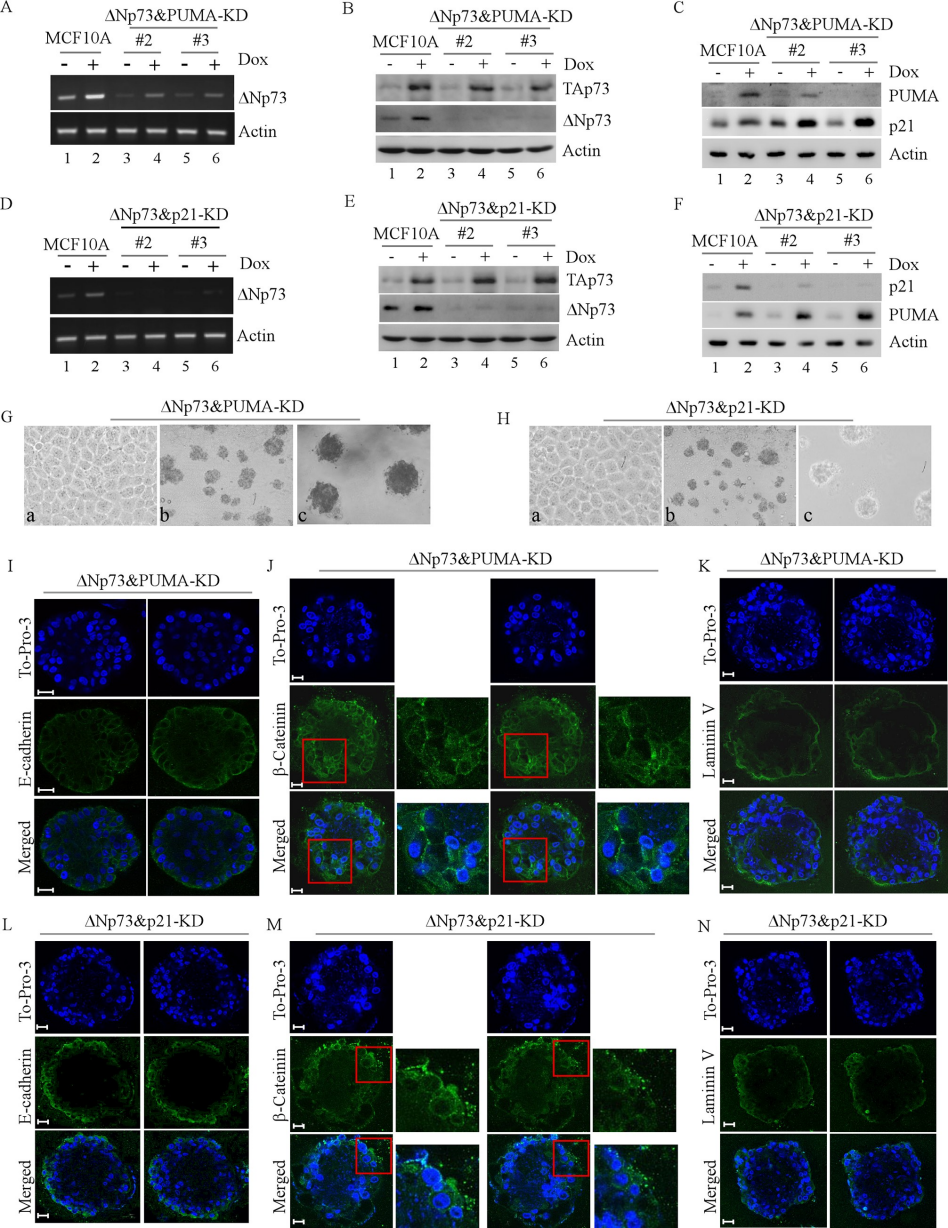
Knockdown of ΔNp73 counters the effect of PUMA-KD or p21-KD on MCF10A cell morphogenesis. **A,** Generation of MCF10A cells in which both ΔNp73 and PUMA were stably knocked down (clones #2 and #3). Parental and ΔNp73&PUMA-KD MCF10A cells were untreated or treated with 0.2 μM doxorubicin for 24 h and total RNAs were collected for RT-PCR to examine the levels of ΔNp73 and actin mRNA. **B-C,** The levels of TAp73 **(B)**, ΔNp73 **(B)**, PUMA **(C)**, p21 **(C)** and actin **(B-C)** proteins were measured in parental and ΔNp73&PUMA-KD MCF10A cells mock-treated or treated with doxorubicin (0.2 μM). The samples were loaded multiple times to detect TAp73, ΔNp73, PUMA, and p21 protein, respectively. Both TAp73 and actin panels were from the same gel whereas ΔNp73 panel was from a different gel. The actin panels were representative ones and used as a loading control. **D,** Generation of MCF10A cells in which both ΔNp73 and p21 were stably knocked down (clones #2 and #3). Parental and ΔNp73&p21 MCF10A cells were untreated or treated with 0.2 μM doxorubicin for 24 h and total RNAs were collected for RT-PCR to examine the level of ΔNp73 and actin mRNA. **E-F,** The levels of TAp73 **(E),** ΔNp73 **(E),** PUMA **(F),** p21 **(F)** and actin **(E-F)** proteins were measured in parental and ΔNp73&p21-KD MCF10A cells treated with or without doxorubicin (0.2 μM). The samples were loaded multiple times to detect TAp73, ΔNp73, PUMA, and p21 protein, respectively. Both TAp73 and actin panels were from the same gel whereas ΔNp73 panel was from a different gel. The actin panels were representative ones and used as a loading control. **G-H,** Representative images of MCF10A cells with ΔNp73&PUMA -KD **(G)** or with ΔNp73&p21-KD **(H)** in 2-D culture (a, 200X) and 3-D culture (b, 40X; c, 100X). **I** and **L,** Representative confocal images of cross-sections through the middle of acini stained with To-Pro-3 and antibody against E-cadherin in MCF10A cells with ΔNp73&PUMA-KD **(I)** or with ΔNp73&p21 -KD **(L)**. **J** and **M,** Representative confocal images of cross-sections through the middle of acini stained with To-Pro-3 and antibody against β-catenin in MCF10A cells with ΔNp73&PUMA-KD **(J)** or with ΔNp73&p21-KD **(M)**. **K** and **N**, Representative confocal images of cross-sections through the middle of acini stained with To-Pro-3 and antibody against laminin V in MCF10A cells with ΔNp73&PUMA-KD **(K)** or with ΔNp73&p21-KD **(N)**. Scale bar, 20 μm.

## Supporting information

S1 File Raw Blot and membrane underlying the ΔNp73 panels of Fig 6B and Fig 6E. (TIF)

S2 File Raw Blot and membrane underlying the Actin panels of Fig 6B and Fig 6E. (TIF)

S3 File Raw Blot and membrane underlying the Tap73 panels of Fig 6B and Fig 6E. (TIF)

## References

[1] Zhang Y, Yan W, Jung YS, Chen X (2013) PUMA Cooperates with p21 to Regulate Mammary Epithelial Morphogenesis and Epithelial-To-Mesenchymal Transition. PLoS ONE 8(6): e66464 10.1371/journal.pone.0066464 23805223 PMC3689819

