# Supplementary figures and images for: Correction: PUMA Cooperates with p21 to Regulate Mammary Epithelial Morphogenesis and Epithelial-To-Mesenchymal Transition
Source: PLoS One. 2020 Aug 7;15(8):e0237624. doi: 10.1371/journal.pone.0237624 (PMC7413548; doi:10.1371/journal.pone.0237624)

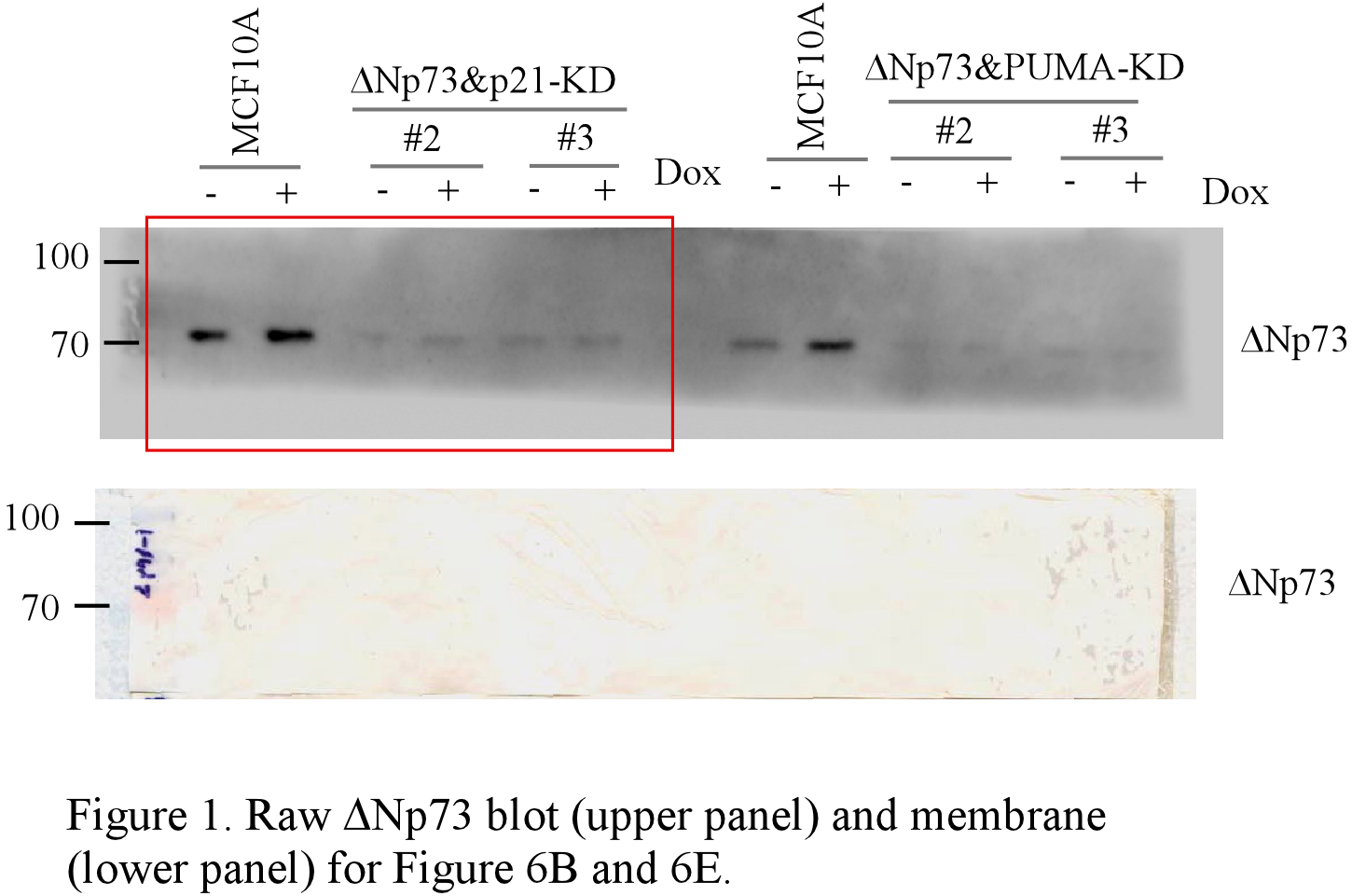

Supplement: S1 File — (TIF) [file pone.0237624.s001.tif]

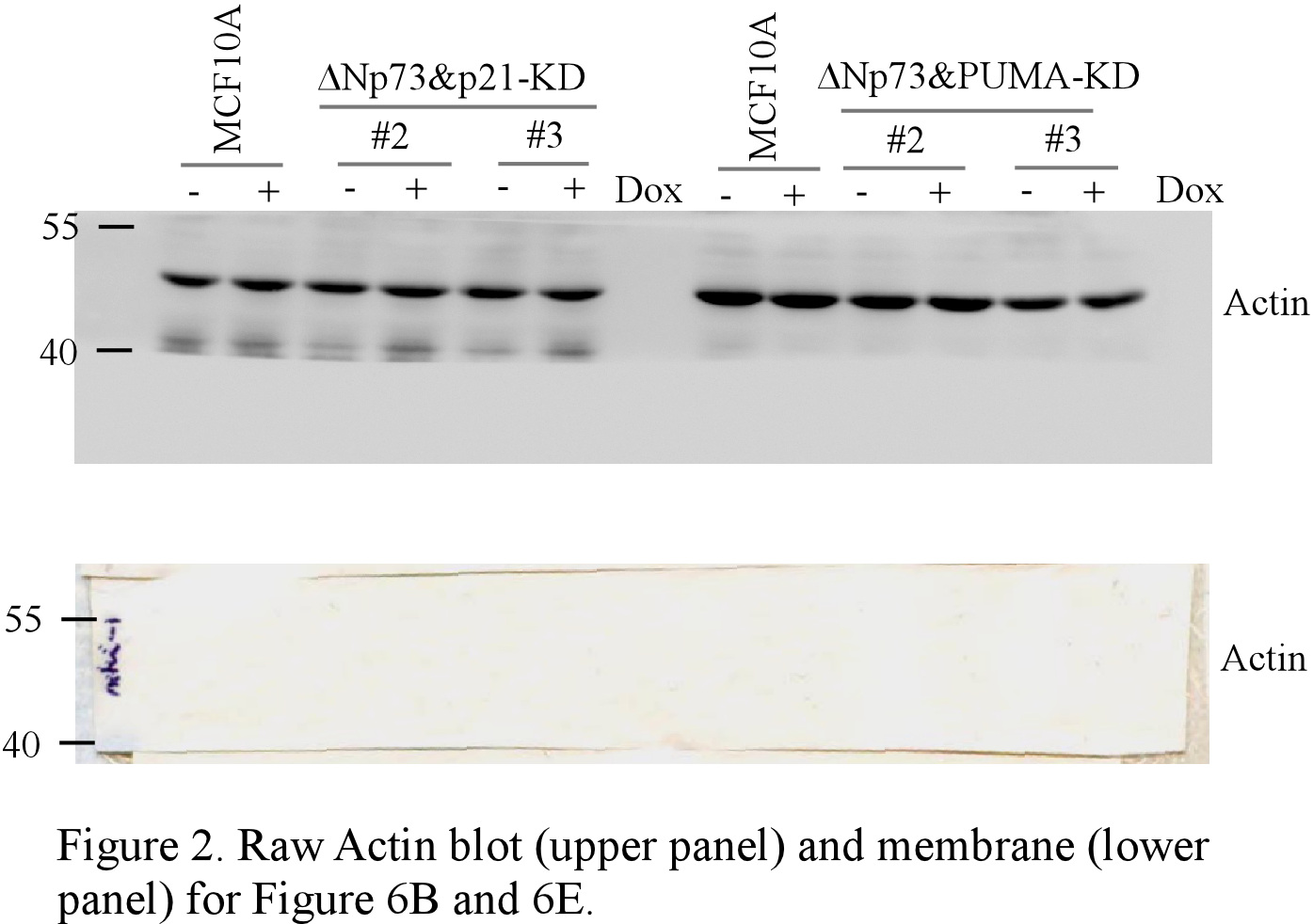

Supplement: S2 File — (TIF) [file pone.0237624.s002.tif]

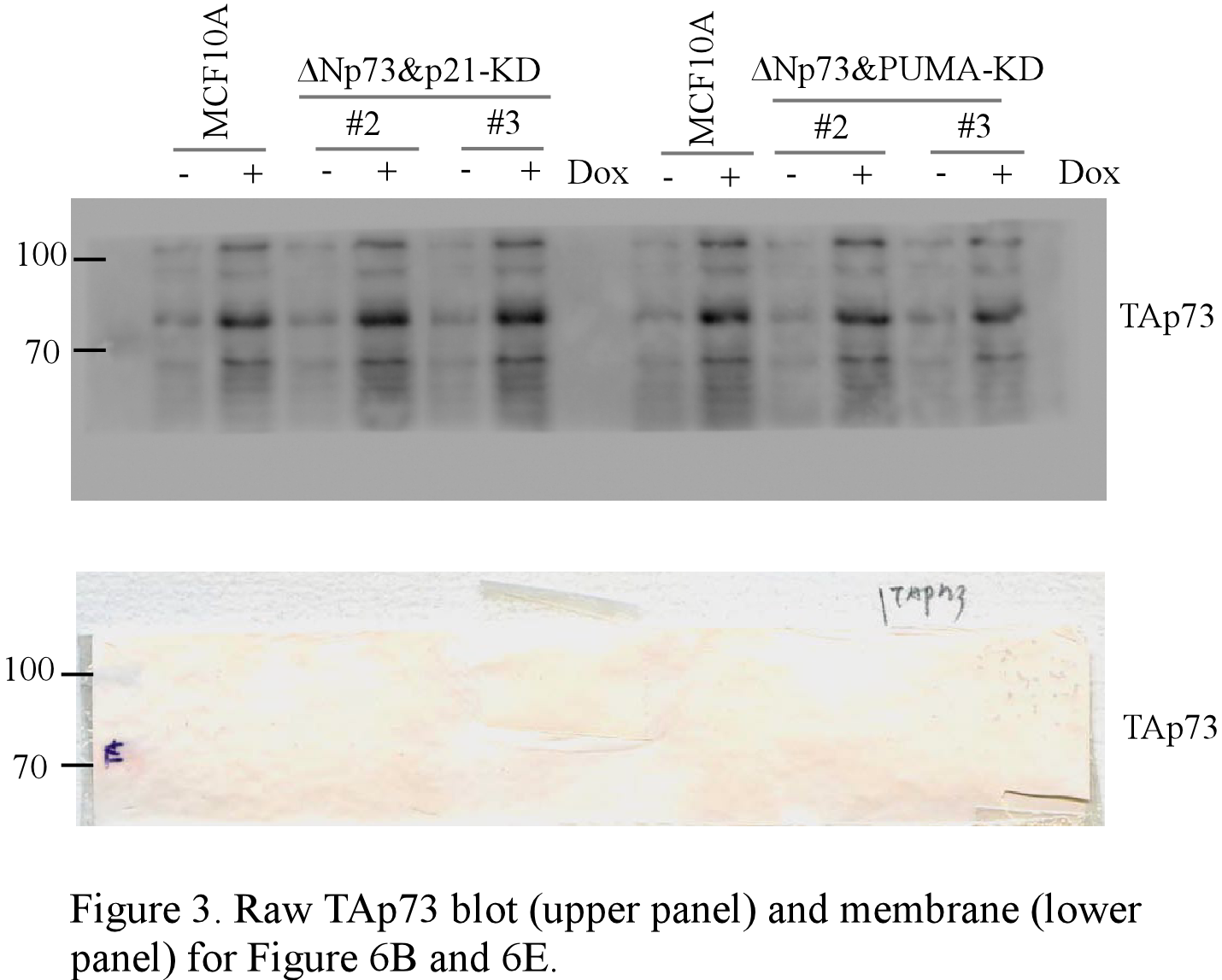

Supplement: S3 File — (TIF) [file pone.0237624.s003.tif]
